# Does task delegation to non-physician health professionals improve quality of diabetes care? Results of a scoping review

**DOI:** 10.1371/journal.pone.0223159

**Published:** 2019-10-11

**Authors:** Sanas Mirhoseiny, Tjarko Geelvink, Stephan Martin, Horst Christian Vollmar, Stephanie Stock, Marcus Redaelli

**Affiliations:** 1 Institute for Health Economics and Clinical Epidemiology, University Hospital of Cologne, Cologne, Germany; 2 Faculty of Medicine, University of Düsseldorf, Düsseldorf, Germany; 3 West-German Center of Diabetes and Health, Düsseldorf Catholic Hospital Group, Düsseldorf, Germany; 4 Department of General Practice, Faculty of Medicine, Ruhr-Universität Bochum, Gebäude MAFO 1/61, Bochum, Germany; 5 Institute of General Practice and Family Medicine, Faculty of Health, Witten/Herdecke University, Witten, Germany; University of Newcastle, AUSTRALIA

## Abstract

**Objective:**

As a result of unhealthy lifestyles, reduced numbers of healthcare providers are having to deal with an increasing number of diabetes patients. In light of this shortage of physicians and nursing staff, new concepts of care are needed. The aim of this scoping review is to review the literature and examine the effects of task delegation to non-physician health professionals, with a further emphasis on inter-professional care.

**Research design and methods:**

Systematic searches were performed using the PubMed, Embase and Google Scholar databases to retrieve papers published between January 1994 and December 2017. Randomised/non-randomised controlled trials and studies with a before/after design that described the delegation of tasks from physicians to non-physicians in diabetes care were included in the search. This review is a subgroup analysis that further assesses all the studies conducted using a team-based approach.

**Results:**

A total of 45 studies with 12,092 patients met the inclusion criteria. Most of the interventions were performed in an outpatient setting with type-2 diabetes mellitus patients. The non-physician healthcare professionals involved in the team were nurses, pharmacists, community health workers and dietitians. Most studies showed significant improvements in glycaemic control and high patient satisfaction, while there were no indications that the task delegation affected quality of life scores.

**Conclusions:**

The findings of the review suggest that task delegation can provide equivalent glycaemic control and potentially lead to an improvement in the quality of care. However, this review revealed a lack of clinical endpoints, as well as an inconsistency between the biochemical outcome parameters and the patient-centred outcome parameters. Given the vast differences between the individual healthcare systems used around the world, further high-quality research with an emphasis on long-term outcome effects and the expertise of non-physicians is needed.

## Introduction

With more than 422 million adults estimated to be affected worldwide [1], diabetes in the 21st century is often considered an epidemic, and it threatens the economies of every nation. Due to an ageing population and lifestyle changes that lead to reduced physical activity and increased obesity, the prevalence of diabetes has been steadily increasing over the past few decades, and is expected to increase further in the future [2–4]. To date, diabetes-related healthcare costs worldwide make up an estimated 12% of overall healthcare expenditure per year [3]—$825 billion in total [5]—with the largest components of costs being related to the treatment of diabetes complications [6, 7]. These microvascular and macrovascular complications can potentially lead to coronary artery disease, strokes, blindness, kidney failure and lower limb amputation [8], and are strongly associated with insufficient control of blood sugar levels [9]. It is known that adequate diabetes treatment can delay or even prevent diabetic complications, thus resulting in great improvements to quality of life and substantial cost savings. However, many patients do not receive adequate treatment, or indeed any treatment at all. Studies have shown that a substantial proportion of those with diabetes remain undiagnosed, since there are often few symptoms during the early years of type-2 diabetes and, when symptoms do manifest, they are not recognised as being related to diabetes [10, 11]. As a result of the lack of symptoms, diabetes patients and those at risk of developing diabetes need to be examined regularly and educated regarding their disease and the importance of rigorous blood sugar control. Previous research has revealed poor levels of diabetes knowledge among patients [11, 12], and that physicians have only limited time to spare for providing diabetes education [13]. Furthermore, diabetes care can be complicated by restricted access to diabetes specialists, especially in underserved rural areas [14].

Due to the complexity of the disease, a variety of physician and non-physician healthcare professionals are involved in the treatment of diabetes patients, including diabetologists, primary care physicians, nurses, dietitians, ophthalmologists, pharmacists, psychologists and podiatrists. In the changing healthcare system landscape, dividing tasks between different healthcare professions could be an effective means of dealing with shortages of physicians and nursing staff. Previous studies have shown that appropriately trained or specialised, non-physician health providers can provide equivalent care to that provided by physicians, resulting in a high level of satisfaction on the patients’ side [15–19]. Stepping away from traditional job demarcations and shifting tasks from physicians to non-physicians could allow us to create new types of work models [20]. The aim of this scoping review is to examine these new types of work models that involve extended roles for non-physicians. Task delegation to non-physician clinicians could potentially free up more time for patient care and allow physicians to focus on more complex medical care issues [21]. This study constitutes the first part of the scoping review, and contains a subgroup analysis that further assesses all the studies that involved team-oriented interventions. Diabetes teams consisting of physician and non-physician health providers could potentially improve quality of care in inpatient and outpatient settings by diminishing the length of stay and thus reducing mortality and costs [22–25].

The objective of this scoping review is to determine whether task delegation in diabetes teams could improve quality of diabetes care. Quality of care is assessed applying the conceptual model by Donabedian, which consists of three quality dimensions: *structure*, *process* and *outcome* [26].

## Methods

### Research design

At the beginning of the literature search process, several methods of systematic reviews were considered, including the meta-analysis approach. Due to the high number of delegation studies identified and their heterogeneity, the purpose of this review should be to identify the types of evidence available and the key concepts and characteristics of task delegation, as well as potential fields of application. For this reason, a scoping review approach was chosen over conducting a systematic review [27]. Scoping reviews can provide a method for mapping evidence from a specific research area by showing existing research findings and revealing gaps in the evidence base at the same time.

Assuming that different countries demonstrate different socialisation processes in the respective health systems, the relationship between individual health personnel and that between health personnel and their patients will be affected by those socialisation processes. These interpersonal relationships might affect the outcomes of studies and lead to a bias that cannot be identified using the data from the studies or based exclusively on knowledge of the health systems. The conservative approach of the scoping review was thus chosen in order to ensure that the results remain reliable.

The conduct of this scoping review is based on the five-stage-approach as described by Arksey and O’Malley, which includes *identifying the research question*, *identifying relevant studies*, *study selection*, *charting the data and collating*, *summarising and reporting the results* [28]. Furthermore, this scoping review was conducted in accordance with the PRISMA Extension for Scoping Reviews (PRISMA ScR) [29].

### Identifying relevant studies

#### Inclusion and exclusion criteria

All studies published between 1994 and 2017 that described a defined task delegation from physicians to non-physicians in the treatment of patients with diabetes mellitus were included. 1994 was chosen as the starting year because it marks the beginning of modern, evidence-based medicine [30]. Interventions with type-1, type-2 and gestational diabetes patients were included in this review. Randomised controlled trials, clinical controlled trials and before/after studies were included, while feasibility trials, abstracts, qualitative studies and study protocols were excluded.

In addition to this, systematic reviews that met the eligibility criteria were identified and, in order to avoid duplicates, the primary literature in these reviews was checked for eligibility and included in the scoping review. In order to be eligible, the studies had to be published in German, English or French language. The initial literature search took place in 2014. Studies from between 1993 and 2013 were included in an economic evaluation, which was published in 2017 [31]. An updated search was conducted in 2018 to include all studies published from 2014 to 2017. Systematic searches were performed using the PubMed, Embase and Google Scholar databases. Due to the lack of consistent keywords, a combination of different keywords was used in order to ensure that all the relevant studies were identified. The literature search was conducted using the keywords *delegation*, *substitution*, *hospital*, *structured care* and *managed care* in combination with the keywords *nurse*, *team*, *dietitian*, *pharmacist*, *community health worker* and *social worker*.

### Study selection

Two reviewers (SM, TG) assessed each potentially relevant study independently. First, the abstracts were reviewed. If the information provided therein suggested that the study met the inclusion criteria, the full articles were then retrieved for further assessment. Duplicates were removed at every stage. Differences between the two reviewers’ results were resolved through discussion, and a third reviewer (MR) was consulted when necessary.

### Charting the data

Two reviewers (SM, TG) independently extracted data from each included study. In detail, the following data was collected from the studies: characteristics of the study population, lengths of intervention and follow-up, use of clinical guidelines, team characteristics, qualification and training of non-physician team members, use of telemedicine, and outcome parameters. The outcome parameters are HbA1c, fasting blood glucose, body weight/body mass index, blood pressure, lipid profile, nephropathy parameters, ‘hard’ clinical endpoints like morbidity and mortality, patient satisfaction, quality of life, diabetes knowledge and cost savings.

## Results

**Fig 1:**
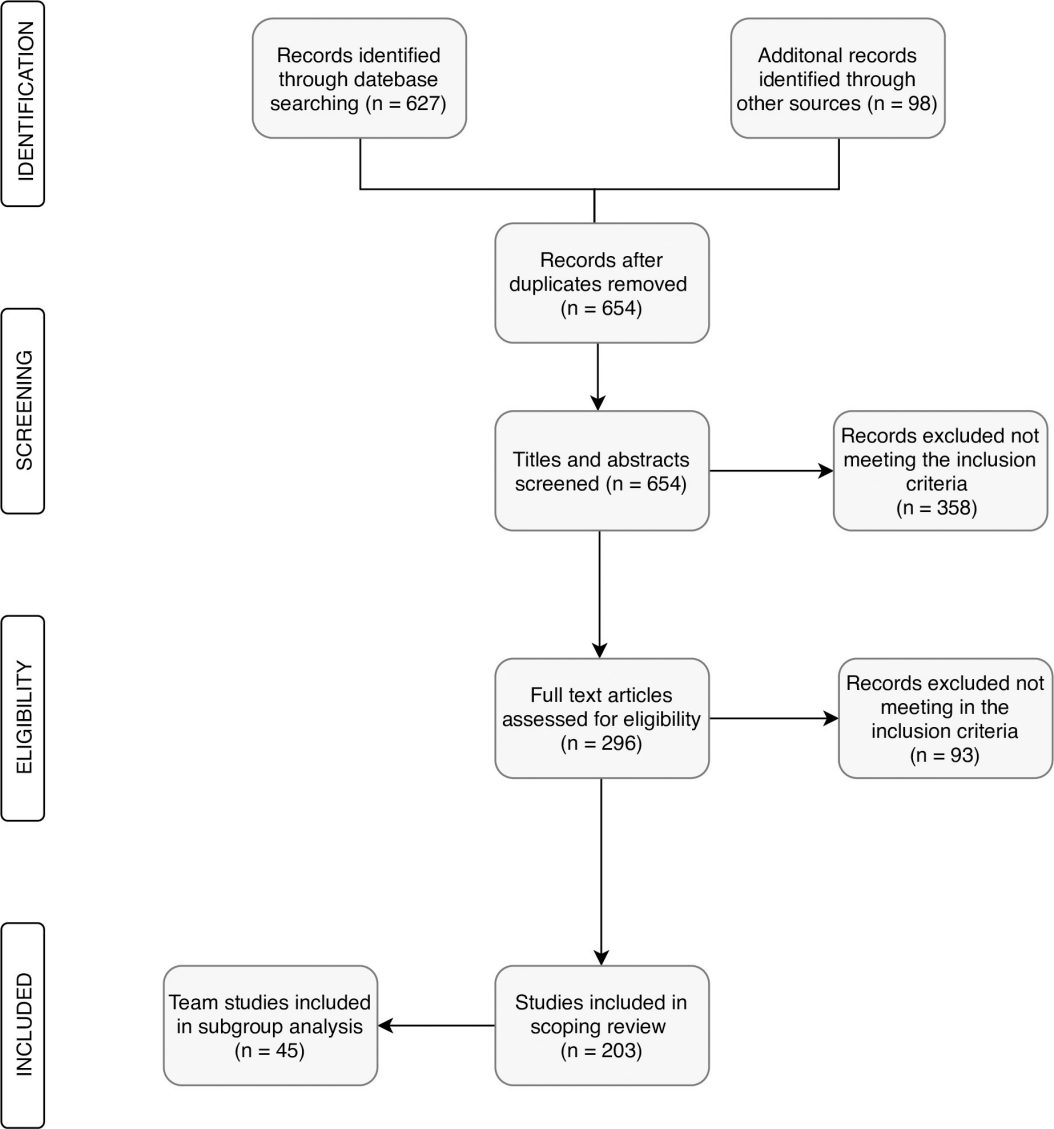
Flowchart for the literature search process.

The initial literature search in 2014 identified 403 potentially relevant studies in the electronic databases; the updated literature search in 2018 identified 224 potentially relevant studies published between 2014 and 2017. A further 98 potential studies were found by means of a manual search, which consisted of checking the reference lists of studies that had already been identified as relevant. Fig 1 shows the flowchart for the search process.

Following the initial screening of the titles and abstracts, 296 full-text articles were examined. A total of 137 studies initially met the eligibility criteria and were included in the scoping review. The updated literature search yielded 66 studies published between 2014 and 2017; these were subsequently included in the updated scoping review. A total of 45 studies with a team-based approach were included in this subgroup analysis.

### Study characteristics

#### Study design and methodology

There were 30 randomised controlled trials (67%), four of which were conducted in a cluster design; seven controlled clinical trials (16%) and 5 before/after studies (11%). Most of the studies indicated inclusion and exclusion criteria (43 studies, 96%) and study limitations (37 studies, 82%). Power and sample size calculations were conducted in 27 of the studies (60%).

#### Study setting

The majority of the studies were conducted in the USA (24 studies, 53%), the Netherlands (5 studies, 11%) and China (3 studies, 7%). Most of the interventions were performed in an outpatient setting (44 studies, 98%); one study (Koproski et al. 1997) was conducted solely in an inpatient setting. Thirty-six of the studies (80%) were performed in a primary care setting where day-to-day healthcare is delivered mainly by primary care physicians. Two of the studies (4%) took place in a hospital-based ambulatory care setting. Six of the studies (13%) included a community care approach.

#### Patient characteristics

A total of 12,092 participants were followed up for a mean duration of 15.88 months, ranging from three to 88 months (see Table 1). A slight majority of the participants were female (52.9%). The participants had a mean age of 56.7 years (standard deviation: 10.6) and a mean diabetes duration of 10.8 years (standard deviation: 4.3). Most of the interventions only included type-2 diabetes patients (42 studies, 93%). There were three studies that only included type-1 diabetes patients (Jansa et al. 2006, Christie et al. 2016 and Clapin et al. 2017), and no studies that included gestational diabetes patients. Table 1 shows the characteristics of the included patients.

**Table 1:** Patient characteristics.

Patient characteristics	
Total number of patients	12,092
Mean duration of follow-up	15.88 months
Mean duration of diabetes	10.8 years
Mean age of participants	56.7 years
Percentage of female patients	52.9%

#### Type of intervention

Most of the team interventions involved diabetes education as an aid to self-management (39 studies, 87%) in group and individual sessions; clinic-based sessions or in-home visits. Twenty-four of the studies (53%) included the use of telemedicine, mostly in the form of telephone calls (79%) and video conferences (21%). The frequency of patient contact ranged from daily contact (face-to-face or via telephone or video conference) to visits every couple of months. Twenty-eight of the studies (62%) indicated that the intervention was based on national or international guidelines. A further seven studies (16%) included predefined protocols or algorithms. Ten of the studies (22%) did not declare any guidelines, protocols or algorithms.

#### Team characteristics

The teams consisted of various non-physician healthcare professionals, including nurses (44 studies, 98%), dietitians (27 studies, 60%), community health workers and social workers (10 studies, 22%), pharmacists (10 studies, 22%), and psychologists (4 studies, 9%). In 24 of the studies, a physician was involved in the team. On average, there were three different healthcare professionals in each team, ranging from teams with only two team-members to teams with more than seven different non-physician providers.

#### Team supervision and qualification

Only ten studies indicated that non-physician healthcare providers were supervised by physician team-members. Twenty-seven studies (60%) reported detailed qualifications for the team members, such as certified diabetes educator, diabetes specialist nurse or nurse case manager.

Six studies (13%) specified that the team members received specific training prior to the intervention. Table 2 provides a summary of all 45 studies included in the subgroup analysis.

**Table 2:** Summary of all 45 studies with team-based interventions (continued).

Main author (Year, country	SD	Pat	F/U	Team	Intervention	Control	Outcomes reported							Main results
							HbA_1c_	FBG	BP	LP	QoL	PS	DK	
**de Sonnaville** **(1997, NL)** **[54]**	CCT	n = 681 T2DM	24	Diabetes nurse educator, dietitian, podiatrist 24/7 supervision by a diabetologist	Patient registration system, consultation facilities of a dietitian nurse, protocolised blood glucose lowering therapy advice which included home blood glucose monitoring with regular telephone contact to adjust insulin dose	Usual care by general practitioner consisting of regular appointments for assessment of glycaemic control and review of complications and cardiovascular risk factors			**X**	**X**		**X**		Significant improvement of HbA_1c_, lipid profiles and diastolic blood pressure as well as a slight improvement of patient satisfaction in the intervention group
**Koproski** **(1997, USA)** **[55]**	RCT	n = 311 N/A	6	Diabetes nurse educator and endocrinologist *(Inpatient setting)*	Diabetes screening for all new admissions in the hospital as well as daily visits by a diabetes team with nutrition and social work consultations if needed	Care from physicians, nurses, nutritionists, and social workers normally received in the medical/surgical units		**X**						Shorter length of stay and fewer readmissions in the intervention group
**Goddijn** **(1999, NL)** **[56]**	B/A	n = 99 T2DM	12	Diabetes specialist nurse, dietitian, ophthalmologist	Intensified control by a diabetes specialist nurse consisting of diabetes education, self-care techniques and dietitian and ophthalmologist appointments	No control group (before/after study design)	**X**				**X**			Significant improvement of HbA_1c_ and quality of life
**Ridgeway** **(1999, USA)** **[57]**	RCT	n = 56 T2DM	12	Diabetes nurse educator and dietitian	Education and behaviour modification sessions in the primary care clinic by a nurse and a dietitian	Not specified/usual care by primary care physician	**X**	**X**		**X**	**X**		**X**	Significant improvement of HbA_1c_, fasting blood glucose, lipid profiles and BMI in the intervention group
**Sadur** **(1999, USA)** **[58]**	RCT	n = 185 T1DM T2DM	6	Diabetes nurse educator, psychologist, nutritionist and pharmacist	Multi-disciplinary outpatient diabetes care management in cluster visit setting with telephone contact in between the visits and individual sessions if needed	Diabetes care by primary care physician	**X**					**X**		Significant improvement of HbA’ in the intervention group, high patient satisfaction
**Wagner** **(2001, USA)** **[59]**	RCT	n = 1.001 N/A	12	Primary care physician, Practice nurse, research, nurse, clinical pharmacist	Individual visits with the diabetes team and group Educational sessions to support self-management	Usual care, not specified	**X**			**X**		**X**		Significantly more recommended preventive Procedures and non-significantly higher patient satisfaction in the intervention group
**Brown** **(2002, USA)** **[60]**	RCT	n = 502 T2DM	12	Nurses, dietitians, community workers	Instructional group sessions on diabetes education and self-management by bilingual nurses, dietitians and social workers	Usual care by private physicians or local clinics	**X**	**X**	**X**	**X**			**X**	Significant reduction in HbA_1c_ and fasting blood glucose, as well as higher diabetes knowledge scores in the intervention group
**Keyserling** **(2002, USA)** **[61]**	RCT	n = 219 T2DM	12	Dietitian and community health worker	Clinic and community-based intervention: Group sessions, individual counselling and regular phone calls from a peer counsellor to reinforce behaviour change goals Clinic intervention only: Educational group sessions and individual counselling to support behaviour changes	Minimal intervention: Educational pamphlets mailed to participants	**X**			**X**			**X**	Primary outcome physical activity significantly higher in clinic and community based intervention group compared to minimal intervention group
**Raji** **(2002, USA)** **[62]**	RCT	n = 317 N/A	12	Physician, nurse, dietitian, pharmacist, exercise physiologist and social worker	Intensive education group: 3.5 days of a structured curriculum education Passive education group: Educational materials by mail every 3 months	Patients who declined participation in the study	**X**							Significant improvement of HbA_1c_ in both the intensive and passive education groups compared to the control group
**Gary** **(2003, USA)** **[63]**	RCT	n = 186 T2DM	12	Nurse and community health worker	Nurse intervention, CHW intervention or combined nurse and CHW intervention consisting of either face-to-face clinic visits or telephone contacts to improve diabetes education and self-management	On-going care from the patients' own healthcare professionals, quarterly newsletter about diabetes-related topics	**X**		**X**	**X**				No significant improvement of HbA’, but of triglyceride and diastolic blood pressure compared to control group
**Izquierdo** **(2003, USA)** **[64]**	RCT	n = 56 T1DM+ T2DM	12	Nurse and dietitian	Educational intervention via videoconferencing	Face-to-face, in-person education	**X**		**X**	**X**	**X**	**X**	**X**	Non-significant improvement of HbA’ in both groups, high patient satisfaction in the telemedicine group
**Litaker** **(2003, USA)** **[65]**	RCT	n = 157 T2DM	12	Nurse and physician	Chronic disease management and use of clinical practice algorithms, patient education on disease self-management strategies, regular monitoring and feedback delivered primarily by the nurse	Usual care by primary care physician as prior to the intervention	**X**		**X**	**X**	**X**	**X**		Significant improvement of HbA’, HDL and patient satisfaction in the intervention group, higher costs for personnel in the intervention group after 1 year
**Majumdar** **(2003, CAN)** **[66]**	B/A	n = 393 T2DM	6	Specialist physicians, nurses, dietitians, pharmacists	Multi-disciplinary diabetes specialist team traveling to rural areas to provide education for primary care physicians and patients	Usual care delivered by local providers with the addition of bimonthly visits by a traveling team raising diabetes awareness and emphasising patient self-management	**X**		**X**	**X**		**X**		Significant improvement of blood pressure and patient satisfaction
**So** **(2004, CHN)** **[67]**	CCT	n = 172 T2DM	88	Research nurse and diabetologist	Structured care consisting of regular medical visits and monitoring by diabetologist and nurse	Regular monitoring by generalists or various specialists	**X**	**X**	**X**	**X**				Significantly lower mortality in the intervention group
**California MediCal Type-2 Diabetes Study Group** **(2004, USA)** **[68]**	RCT	n = 362 T2DM	36	Nurse and dietitians in close cooperation with endocrinologist	Blood glucose, hypertension and dyslipidaemia management, education measures regarding diet, exercise and self-care behaviours, retinopathy, nephropathy and cardiovascular disease prevention	Traditional primary care treatment	**X**		**X**	**X**				Significant and sustainable improvement of HbA_1c_ in the intervention group
**Maislos** **(2004, Israel)** **[69]**	RCT	n = 82 T2DM	6	Diabetes nurse educator, dietitian, diabetologist	Diabetes education in order to improve compliance and lifestyle changes	Usual treatment provided by doctors and nurses	**X**	**X**						Significant improvement of HbA_1c_ and fasting blood glucose as well as significant changes in antidiabetic medications in the intervention group
**O'Hare** **(2004, UK)** **[70]**	cRCT	n = 361 T2DM	12	Community health worker and diabetes specialist nurse	Diabetes education provided by nurses and multi-lingual community health workers to enhance patient understanding and compliance	Diabetes care with existing practice resources	**X**		**X**	**X**				No significant change in HbA_1c_ and no difference between the intervention and control group; intervention group had significant reduction in diastolic blood pressure
**Philis-Tsimikas** **(2004, USA)** **[71]**	CCT	n = 310 T1DM+ T2DM	12	Medical assistant, dietitian, certified diabetes nurse educator	Group education and nurse case management consisting of reviewing of self-monitored blood glucose results, recommendation of medication changes and order of follow-ups and return visits	Usual treatment	**X**		**X**	**X**		**X**	**X**	Significant improvement of HbA_1c_, lipid profile and blood pressure compared to the control group. Significant improvement of diabetes knowledge and patient satisfaction
**Dijkstra** **(2005, NL)** **[72]**	cRCT	n = 997 T1DM+ T2DM	12	Diabetes specialist nurse and internist	Educational meetings for professionals and patients, as well as the introduction of a diabetes passport	Usual care	**X**		**X**	**X**	**X**	**X**		Significant changes in HbA_1c_ compared to the control group, no changes in quality of life measures and patient satisfaction
**Keers** **(2005, NL)** **[73]**	B/A	n = 410 T1DM+ T2DM	12	Diabetes nurse specialist, endocrinologist, dietitian, social worker, psychologist, physiotherapist, occupational therapist, activity therapist	Multi-disciplinary intensive diabetes education program on self-management strategies consisting of group sessions and individual support	Non-referred outpatients from the diabetes clinic	**X**				**X**			Significant improvement of HbA_1c_ and significant reduction in costs
**Rothman** **(2005, USA)** **[74]**	RCT	n = 217 T2DM	12	Pharmacist and diabetes care coordinator	Intensive education and counselling, as well as medication management through face-to-face visits in the clinic and telephone contact in close cooperation with primary care physician	Usual care by primary care provider	**X**		**X**	**X**		**X**	**X**	Significant improvement of HbA_1c_ and blood pressure as well as improvement of diabetes knowledge and treatment satisfaction in the intervention group
**Taylor** **(2005, CAN)** **[75]**	RCT	n = 66 T2DM	4	Nurse and dietitian	Home visits by a nurse, a dietitian and if wanted an exercise specialist providing diabetes education and patient self-awareness	Standard medical care consisting of office visits at 3-month intervals	**X**	**X**	**X**	**X**	**X**	**X**		Small but non-significant improvement of HbA_1c_, blood pressure, cholesterol and quality of life parameters in the intervention group, and high patient satisfaction
**Jansa** **(2006, ESP)** **[76]**	RCT	n = 40 T1DM	12	Team members not specified (except for a diabetes nurse)	Electronic (telematic) transfer of glycaemic values with feedback by diabetes team	Conventional care	**X**				**X**	**X**		Significant improvement of glycaemic control in both groups, lower costs in the intervention group, longer telematic consultations due to technical difficulties
**Chan** **(2009, CHN)** **[77]**	RCT	n = 205 T2DM	24	Diabetologists, endocrine trainees, diabetes nurse, dietitian	Structured care using a case report book containing predefined scheduled visits, assessment items and predefined treatment targets	Usual care (either in a diabetes clinic by a diabetes team or a general medical clinic by non-diabetes specialist or an internist)	**X**		**X**	**X**				Significant improvement of HbA_1c_ and diastolic blood pressure; significantly more patients reached at least three treatment goals
**Gary** **(2009, USA)** **[78]**	RCT	n = 542 T2DM	36	Nurse and community health worker	1) Minimal intervention: Telephone based, executed by a lay health educator to empower patients’ involvement in their healthcare 2) Intensive intervention: Education and follow-up services of a nurse case manager and community health worker team to improve self-management	No control group without any intervention	**X**		**X**	**X**				Significant improvement of HDL and diastolic blood pressure in the intensive intervention group, significant decline of HbA’ in those groups who had more visits with nurse and community health worker, when compared with the minimal group
**Kim** **(2009, USA)** **[79]**	RCT	n = 83 T2DM	11	Nurse and dietitian	Education program to enhance diabetes knowledge and promote self-care behaviours; home glucose monitoring with teletransmission, and regular telephone counselling by a bilingual nurse	Delayed intervention	**X**	**X**		**X**	**X**		**X**	Significant improvement of HbA_1c_, fasting blood glucose, lipid levels, diabetes knowledge and quality of life in the intervention group
**Whittemore** **(2009, USA)** **[80]**	RCT	n = 58 T2DM	6	Nurse and dietitian	Lifestyle change program including education on nutrition, prevention and exercise, as well as behavioural and motivational interviews	Enhanced standard care (written information about diabetes prevention, individual sessions with nurse practitioner and dietitian)		**X**		**X**		**X**		Significant improvement of lifestyle behaviours (nutrition, exercise) and patient satisfaction in the intervention group, no significant improvement in clinical outcome parameters
**Edelman** **(2010, USA)** **[81]**	RCT	n = 239 T2DM	12	Internist, pharmacist and nurse or certified diabetes educator	Educational group sessions, individualised treatment plans for medication or lifestyle; telephone contact between group sessions	Usual care, without any active intervention	**X**		**X**					Significant improvement of systolic blood pressure, non-significant improvement of HbA_1c_, significantly fewer emergency care visits in the intervention group
**Mayes** **(2010, USA)** **[82]**	B/A	n = 19 T2DM	42	Nurse, community health worker, primary care physicians and endocrinologists	Regular home visits, mail/phone contact and videoconferencing sessions with expert medical team (nurses and endocrinologists) and bilingual community health worker with a central role as mentors and peer-educators	No control group (before/after study design)	**X**		**X**					Significant improvement of HbA_1c_
**Fokkens** **(2011, NL)** **[83]**	CCT	n = 1,001 T2DM	36	Diabetes specialised nurse, practice nurse, dietitian and general practitioner	Education program for both patients and healthcare professionals, introduction of a diabetes passport and structured registration program used for comparisons within and between practices	Usual care consisting of four checks a year	**X**		**X**	**X**			**X**	Significant long-term improvements in blood pressure and cholesterol
**Naik** **(2011, USA)** **[84]**	RCT	n = 87 T2DM	3	Nurse, dietitian, generalist	Group and individual sessions on diabetes education and self-management strategies including feedback on treatment goals	Group and individual sessions by a diabetes nurse educator							**X**	Significantly better understanding of diabetes and target goals in the intervention group
**Weinger** **(2011, USA)** **[85]**	RCT	n = 222 T1DM+ T2DM	12	Nurse and dietitian	Structured behavioural arm: Education sessions concentrating on self-care behaviour strategies and goal-setting-techniques	Group attention control group: Group sessions with homework; no training in cognitive behaviour strategies or structured goal-setting-activities Individual control group: Unlimited, voluntary one-on-one appointments with nurse and dietitian	**X**				**X**			Significant improvement of HbA_1c_ and quality of life scores in all three groups. Greater improvement of HbA1c in the structured behavioural arm
**Chan** **(2012, CHN)** **[86]**	RCT	n = 120 T2DM	9	Pharmacist and nurse	Face-to-face interviews with pharmacist before each physician visit about importance of medication adherence, drug knowledge, skills, perceived health and cognitive functions	Usual care without pharmacist intervention	**X**		**X**	**X**			**X**	Significant reduction in HbA_1c_ and coronary heart disease risk compared to the control group
**Debussche** **(2012, REU)** **[87]**	RCT	n = 398 T2DM	12	Nurse and dietitian	Inpatient educational sessions, followed by quarterly outpatient visits by nurses and dietitians; postal and telephone reminders of scheduled visits	Just one educational visit one year after initial hospitalisation	**X**	**X**	**X**	**X**				Significant improvement of HbA_1c_, blood pressure and nutrition outcomes in both groups; no significant differences in any outcomes between the two groups
**McFarland** **(2012, USA)** **[88]**	CCT	n = 103 T2DM	6	Pharmacist and nurse	Face-to-face visits between pharmacist and patients followed by clinical decisions of the pharmacist; use of a messaging device to communicate in between the visits. nurse was responsible for contacting the patient and evaluating specific health concerns	Face-to-face visits with pharmacist, with telephone calls in between the visits. no involvement of a nurse: the pharmacist alone evaluated specific healthcare concerns	**X**							Non-significant reduction in HbA_1c_ in both groups with significantly more patients reaching HbA_1c_ target goals in the intervention group
**Toledo** **(2012, USA)** **[89]**	B/A	n = 25 T1DM+ T2DM	4.5	Nurse and endocrinologist	Teleconsultation via videoconferencing between an urban endocrinology office and a rural clinic office to convey medical interviews, laboratory data review and treatment recommendations; a nurse ensured coordinating between the two sites	No control group (before/after study design)	**X**					**X**		Significant improvement of HbA_1c_ and high levels of satisfaction on patients and provider’s side
**DePue** **(2013, USA)** **[90]**	cRCT	n = 268 T2DM	12	Nurse and community health worker	Group sessions and individual home visits by a nurse/community health worker to improve diabetes self-management; if necessary feedback to physicians about patient care needs	Wait-list group (community health worker intervention after 1 year)	**X**		**X**					Significant improvement of HbA_1c_ in the intervention group
**Levin** **(2013, DNK)** **[91]**	CCT	n = 81 T1DM+ T2DM	55	Nurse and physician	Telemedical consultations with patient and nurse on an island in audio-visual contact with a physician on mainland	Results were compared to data from the Danish National Diabetes Registry (37.567 patients)	**X**		**X**	**X**		**X**		Significant reduction in HbA_1c_, high patient satisfaction
**Russell** **(2013, AUS)** **[92]**	CCT	n = 373 T2DM	12	General practitioner, endocrinologist, diabetes nurse educator, psychologist, podiatrist, dietitian	Initial screening by a nurse followed by a management plan developed by a GP in consultation with an endocrinologist. Regular phone contact between patients and nurse for insulin stabilisation, motivation and problem-solving	Usual care by an endocrinologist at the diabetes clinic as well as a group education session by a diabetes nurse educator, dietitian and podiatrist	**X**		**X**	**X**				Significant improvement of blood pressure and lipid levels in the intervention group compared to the control group; notable HbA_1c_ reduction in both groups
**Liou** **(2014, TWN)** **[93]**	RCT	N = 95 T2DM	6	Nurse, dietitian, diabetic specialist, primary physician	Six-session diabetes intervention consisting of in person internet sessions providing the patient with diabetic education as well as interactive videoconferencing by a shared care team	Usual care groups received one diabetes education session conducted individually by a licensed practical nurse	**X**		**X**	**X**				Significant reduction in HbA_1c_ in the intervention group, no significant differences in lipid profiles and blood pressure between the groups
**Chen** **(2016, TWN)** **[94]**	RCT	n = 100 T2DM	6	Pharmacist, physicians, certified diabetes educator nurses, dietitians	Assessment of medication adherence, appropriateness of the current medication regimens and regular follow-up visits (monthly telephone calls and face-to-face visits if required), screening for depression, diabetes education, recommendations to the patients’ physicians, and referral of patients to other diabetes care team members	Standard care without pharmacist intervention	**X**							Significant reduction in HbA_1c_ in the intervention group, compared to HbA_1c_ increase in the control group, no significant differences in medical expenses and hospitalisation rates between the groups
**Christie** **(2016, UK)** **[95]**	cRCT	N = 362 T1DM	24	Paediatric specialist nurse with one other team member (trained staff, nurse, dietitian, psychologist)	Group education programme for children with diabetes and their families consisting of four monthly modules about self-management skills	Regular clinic visits to normal clinics and appointments with nursing staff and other clinic staff as clinically indicated or requested by families	**X**				**X**		**X**	No significant improvement of HbA_1c_, diabetes knowledge or quality of life in the intervention group
**Clapin** **(2017, AUS)** **[96]**	RCT	n = 50 T1DM	12	Diabetes nurse educator, dietitian, social worker	Children with newly diagnosed T1DM were discharged after 2 days for home-based management consisting of home visits by nurses and a multi-disciplinary team for two weeks after discharge	Standard inpatient care (5 to 6 day initial inpatient stay)	**X**				**X**	**X**	**X**	No difference between the groups in HbA_1c_, no significant difference in quality of life scores, overall high patient satisfaction in both groups
**Garg** **(2017, USA)** **[97]**	RCT	n = 151 T2DM	12	Nurse and endocrinologist	Diabetes patients undergoing an elective surgery received weekly to monthly phone calls after discharge from a diabetes specialist nurse in collaboration with an endocrinologist. The nurse reviewed patients’ blood glucose values, counselled regarding diet and exercise and made insulin dose adjustments independently after initial approval of the provider	Patients were advised to follow up with their prior diabetes care providers without any interference from the study team	**X**	**X**	**X**	**X**				No significant difference in HbA_1c_ reduction or changes in weight, BMI, blood pressure, lipids levels and renal function between the two groups
**Siaw** **(2017, SGP)** **[98]**	RCT	n = 411 T2DM	6	Physician, clinical pharmacist, dietitian, diabetes nurse educator	Regular follow-up by pharmacists via face-to-face visits or phone calls in addition to usual care	Usual care with referrals to nurses and dietitians as needed	**X**		**X**	**X**		**X**		Significant reduction in HbA_1c_ and significant higher patient satisfaction in the intervention group, no significant improvement of blood pressure and LDL cholesterol in both groups

SD: Study design; Pat Patients; F/U: Length of follow-up in months; T1DM: Type-1 Diabetes mellitus; T2DM: Type-2 Diabetes mellitus; RCT: Randomised controlled trial; cRCT: Cluster-randomised controlled trial; CCT: Clinical trial (non-randomised); B/A: Before/after; study design; GP: General practitioner; FBG: Fasting blood glucose; LP: Lipid profile; BP: Blood pressure; QoL: Quality of life; PS: Patient satisfaction. DK: Diabetes knowledge; N/A: Not available

### Intervention outcomes

#### HbA1c

Almost all of the studies (42 studies, 93%) measured glycaemic control in the form of HbA1c. Thirty of the studies (67%) showed a significant improvement in HbA1c, either in total or compared to the control group. Eight of the studies (18%) resulted in either non-significant HbA1c improvements or no changes in HbA1c, and only four of the studies (9%) showed a non-significant increase in HbA1c. Significant HbA1c increases were not observed in any of the studies. A detailed record of all the HbA1c changes is shown in Table 3.

**Table 3:** HbA1c values and changes after the establishment of an inter-professional team.

Main author (year, country)	INTERVENTION GROUP		CONTROL GROUP		HbA_1c_
	Baseline	Post-follow-up	Baseline	Post-follow-up	
**de Sonnaville [54]** (1997, NL)	7.4 (σ 1.6)	7.0 (σ 1.3)	7.4 (σ 1.9)	7.6 (σ 1.5)	**D**
**Koproski [55]** (1997, USA)	No measurement of HbA_1c_				
**Goddijn [56]** (1999, NL)	10.4 (σ 2.7)	7.8 (σ 1.5)	No control group		**D**
**Ridgeway [57]** (1999, USA)	12.28 (σ 0.72)	11.52 (σ 0.72)	12.26 (σ 0.4)	11.64 (σ 0.4)	**D**
**Sadur [58]** (1999, USA)	9.48	8.18	9.55	9.33	**D**
**Wagner [59]** (2001, USA)	7.5	7.9	7.4	7.9	**I**
**Brown [60]** (2002, USA)	11.81 (σ 3.0)	10.89 (σ 2.56)	11.22 (σ 2.77)	11.64 (σ 2.85)	**D**
**Keyserling [61]** (2002, USA)	2 groups: 10.7 (σ 0.3) 11.0 (σ 0.4)	2 groups: 10.8 (σ 0.4) 10.9 (σ 0.5)	1 group: 11.3 (σ 0.3)	1 group: 10.7 (σ 0.4)	**U**
**Raji [62]** (2002, USA)	9.9 (σ 1.3)	8.0 (σ 1.8)	9.8 (σ 1.2)	8.6 (σ 1.8)	**D**
**Gary [63]** (2003, USA)	3 groups: 8.8 (σ 2.2) 8.4 (σ 2.0) 8.6 (σ 1.9)	3 groups: - 0.31 (σ 0.49) - 0.30 (σ 0.48) - 0.80 (σ 0.52)	8.5 (σ 2.0)	No data available	**U**
**Izquierdo [64]** (2003, USA)	8.7 (σ 2.1)	7.8 (σ 2.2)	8.6 (σ 1.6)	7.6 (σ 1.3)	**D**
**Litaker [65]** (2003, USA)	8.4 (σ 1.4)	- 0.63 (σ 1.5)	8.5 (σ 1.6)	- 0.15 (σ 1.0)	**D**
**Majumdar [66]** (2003, CAN)	7.17 (σ 1.48)		7.59 (σ 1.67)		**U**
**So [67]** (2004, CHN)	7.2 (σ 2.2)	7.6 (σ 1.3)	8.2 (σ 1.6)	7.4 (σ 1.7)	**I**
**Calif. SG [68]** (2004, USA)	9.54 (σ 0.12)	7.66 (σ 0.17)	9.66 (σ 0.13)	8.53 (σ 0.2)	**D**
**Maislos [69]** (2004, ISR)	11.6 (σ 1.3)	9.8 (σ 1.9)	11.1 (σ 1.1)	10.8 (σ 1.6)	**D**
**O'Hare [70]** (2004, UK)	7.8 (σ 1.9)	-0.23 (σ 1.42)	8.1 (σ 2.1)	-0.2 (σ 1.54)	**U**
**Philis-Tsimikas [71]** (2004, USA)	11.8 (σ 1.78)	8.3 (σ 1.7)	11.5 (σ 1.73)	10.4 (σ 2.5)	**D**
**Dijkstra [72]** (2005, NL)	8.1 (σ 1.3)	7.8 (σ 0.07)	8.0 (σ 1.2)	8.2 (σ 0.05)	**D**
**Keers [73]** (2005, NL)	8.5 (σ 1.3)	8.1 (σ 1.2)	No control group	Reference group: 8.0 (σ 1.2)	**D**
**Rothman [74]** (2005, USA)	11.0 (σ 2.0)	-2.5	11.0 (σ 3.0)	-1.6	**D**
**Taylor [75]** (2005, CAN)	7.69	7.40	7.69	8.41	**U**
**Jansa [76]** (2006, ESP)	8.4 (σ 1.2)	Post-F/U: 7.5 (σ 1.4) Post-study: 7.6 (σ 0.9)	8.9 (σ 1.3)	Post-F/U: 7.7 (σ 0.9) Post-study: 7.6 (σ 0.7)	**D**
**Chan [77]** (2009, CHN)	8.2 (σ 1.9)	7.3 (σ 1.3)	8.4 (σ 0.2)	8.0 (σ 1.6)	**D**
**Gary [78]** (2009, USA)	7.7 (σ 2.1)	-0.2 (σ 1.7)	8.0 (σ 2.2)	-0.08 (σ 1.93)	**D**
**Kim [79]** (2009, USA)	9.4 (σ 1.5)	-1.3 (σ 1.3)	9.1 (σ 1.3)	-0.4 (σ 1.4)	**D**
**Whittemore [80]** (2009, USA)	No measurement of HbA_1c_				
**Edelman [81]** (2010, USA)	9.2 (σ 1.4)	8.3	9.2	8.6	**U**
**Mayes [82]** (2010, USA)	9.6	7.2	No control group		**D**
**Fokkens [83]** (2011, NL)	6.5 (σ 1.1)	+0.2 (σ 1.0)	6.9 (σ 1.2)	+0.2 (σ 1.4)	**I**
**Naik [84]** (2011, USA)	No measurement of HbA_1c_				
**Weinger [86]** (2011, USA)	9.12 (σ 1.1)	8.45 (σ 1.3)	2 groups: 9.09 (σ 1.2) 8.90 (σ 1.1)	2 groups: 8.60 (σ 1.3) 8.69 (σ 1.3)	**D**
**Chan [86]** (2012, CHN)	9.7 (σ 1.4)	-1.57 (σ 1.5)	9.5 (σ 1.8)	-0.4 (σ 1.19)	**D**
**Debussche [87]** (2012, REU)	10.0 (σ 2.2)	8.2 (σ 1.6)	10.3 (σ 2.2)	8.3 (σ 1.5)	**D**
**McFarland [88]** (2012, USA)	9.0 (σ 1.5)	6.9 (σ 1.0)	9.1 (σ 1.6)	7.5 (σ 1.1)	**D**
**Toledo [89]** (2012, USA)	9.6	7.2	No control group		**D**
**DePue [90]** (2013, USA)	Table: 9.6 (σ 2.1) Text: 9.8 (σ 2.2)		10.0 (σ 2.3)	10.0 (σ 2.3)	**D**
**Levin [91]** (2013, DEN)	T1DM: 8.7 (σ 0.5) T2DM: 7.9 (σ 0.5)	T1DM: 8.0 (σ 0.6) T2DM: 7.4 (σ 0.3)	Data from National register T1DM: 7.9 T2DM: 7.6	Data from National register T1DM: 7.9 T2DM: 7.6	**D**
**Russell [92]** (2013, AUS)	8.6 (σ 1.9)	7.7 (σ 3.8)	7.9 (σ 1.9)	7.5 (σ 3.5)	**D**
**Liou [93]** (2014, TWN)	8.3 (σ 1.2)	7,6 (σ 1.1)	8.1 (σ 1.2)	8.1 (σ 1.3)	**D**
**Chen [94]** (2016, TWN)	9.22 (σ 1.7)	8.39 (σ 1.2)	8.94 (σ 1.5)	9.37 (σ 1.5)	**D**
**Christie [95]** (2016, UK)	9.9 (σ 1.5)	10.1 (σ 1.9)	10.0 (σ 1.5)	10.0 (σ 1.7)	**I**
**Clapin [96]** (2017, AUS)	11.9 (σ 1.9)	7.4 (σ 0.3)	12.7 (σ 1.7)	7.2 (σ 0.2)	**U**
**Garg [97]** (2017, USA)	8.9 (σ 1.0)	8.2 (σ 1.4)	9.2 (σ 1.1)	8.5 (σ 1.5)	**U**
**Siaw [98]** (2017, SGP)	8.6 (σ 1.5)	8.1 (σ 1.3)	8.5 (σ 1.4)	8.5 (σ 1.4)	**D**

Calif. SG: California MediCal Type-2 Diabetes Study Group; T1DM: Type-1 diabetes mellitus; T2DM: Type-2 diabetes mellitus; Post F/U: Post follow-up. D: decrease (significant); I: increase (non-significant); U: unchanged (decrease non-significant)

#### Patient satisfaction

Patient satisfaction was measured in 16 of the studies. Most of these studies utilised the diabetes treatment satisfaction questionnaire developed by Bradley [32] to measure patient satisfaction. One study (Toledo et al. 2012) measured both patient and provider satisfaction. In summary, satisfaction with team-based care was generally high, with 14 studies showing improvements.

#### Quality of life

Quality of life measurements were performed in 12 of the studies. Most of the studies utilised the SF-36 health survey developed by the RAND cooperation in the Medical Outcomes Study [33]. Only one study (Kim et al. 2009) reported significant improvements in the quality of life scores. The rest of the studies reported non-significant improvements in the quality of life scores, no score differences after the intervention, or no differences between the intervention and control group.

#### Other outcome parameters

Only ten of the studies (22%) conducted an economic evaluation of the team-based care. Six of those studies reported cost savings following the introduction of a diabetes team. The cost savings ranged from $66 to $950 per person, per year.

A more detailed description of all the cost studies included in this scoping review has been published elsewhere [31].

Other outcome parameters included:

- *Fasting blood glucose* (measured in 10 studies, with 3 studies showing improvement in the outcome parameter)
- *Blood pressure* (measured in 26 studies, with 11 studies showing improvement in the outcome parameter)
- *Body mass index/body weight* (measured in 21 studies, with 2 studies showing improvement in the outcome parameter)
- *Lipid profiles* (measured in 25 studies, with 9 studies showing improvement in the outcome parameter)
- *Nephropathy parameters* such as measurements of *creatinine*, *proteinuria* or *glomerular filtration rate* (measured in 8 studies, with no studies showing improvement in the outcome parameter)

## Discussion

The aim of this scoping review is to examine the effects of task delegation to non-physician healthcare providers. A total of 203 highly heterogeneous studies were identified and analysed further in the form of subgroups. This review is a subgroup analysis of 45 studies, with a further emphasis on team-based diabetes care.

Both inpatient and outpatient studies involving type-1 and type-2 patients were included in order to take into account different settings and age groups. Studies involving gestational diabetes patients were also included in order to factor in different gender distributions; however, none of the studies involving gestational diabetes patients met all of the eligibility criteria.

### Evaluating the quality of care

#### Structure

Structure is defined by all the factors that affect the setting in which care is provided, and includes facilities and equipment as well as the qualification and training of medical staff. Our study reveals a lack of detailed reporting on the qualifications and expertise of non-physicians. A total of 18 studies (40%) did not specify qualifications in terms of either basic and supplementary training, or duration and type of professional experience.

The qualifications of non-physician healthcare providers play a very important role in the safety of the delegation process, and are highly relevant when it comes to implementing diabetes teams that involve extended roles for non-physician health professionals.

Twenty-four of the studies (53%) reported the use of telecommunication and information technology, mostly in the form of telephone calls (79%) and video conferences (21%). The application of telemedicine was very heterogeneous, and ranged from contacting patients in isolated, rural regions to regular patient contact in outpatient settings, as well as non-physicians facilitating contact with physician supervisors. This scoping review analysed the use of telemedicine in task delegation contexts. As such, it is not possible to draw any conclusion regarding the sole effect of telemedicine on patient outcomes.

However, previous research has shown that telemedicine can potentially lead to improvements in quality of life, reductions in lethality, and early detection of diabetic complications [34]. While Marcolino et al. found telemedicine to have a positive effect on HbA1c [35], other studies indicate less clear results [36–38]. The differences in the results of the studies may be explained by the differences in the type of technology used and the context of its use. The results of this scoping review show that telemedicine has the potential to support both communication between team-members and patient communication, which could help improve safety and the acceptance of delegation among all the parties involved. Depending on the type of technology used, telemedicine may require costly investments, and its use should be considered on a case-by-case basis, especially in light of an aging population.

#### Process

Another approach to assessing the quality of care is to analyse the care procedure itself, which includes justification of the diagnosis and the therapy. The assessment of medical care procedures is very difficult in practice. In most of the studies included in this scoping review (78%), the care provided was based on evidence-based guidelines or predefined protocols or algorithms. Unfortunately, many of the studies do not specify whether the interventions are based on national or international guidelines.

Previous research has shown that algorithms, protocols and guidelines seem to be a key factor in task delegation [39]. Due to national legal constraints placed on their decision-making, non-physician health providers–and non-medical staff in particular–tend to follow protocols rigorously, which has a positive effect on medical care [40].

#### Intervention outcomes

The vast majority of the studies included in this review showed a significant improvement in glycaemic control following the intervention; HbA1c improvements were either found between the intervention and control groups or before and after the team intervention.

Since only four out of the 45 studies showed non-significant HbA1c increases and none of the studies showed significant HbA1c increases, task delegation could probably enhance effective diabetes management. However, the results of the studies do not necessarily indicate that task delegation and inter-professional care lead to an improvement in HbA1c. This is because firstly, most of the studies did not include long-term observations of HbA1c levels, and secondly, many of the studies included patients with poorly controlled blood sugar levels. As such, analysis of these results could lead to an overestimation of the effect of the team intervention, and this effect may seem more modest if the studies were to include more patients with adequately controlled blood sugar levels.

The patients were generally very satisfied with the team intervention, as it often led to an increase in the time spent with the patients and an emphasis on self-management techniques and individualised education. In summary, patient satisfaction was linked to quantity and quality of contact with healthcare providers [41].

Quality of life scores were not positively influenced by the team intervention in most of the studies included in the review. This may be explained by the fact that diabetes patients are generally known to have a lower quality of life than non-diabetes patients [42].

Furthermore, the patients included in the studies had been suffering from diabetes for a long time (mean diabetes duration: 10.8 years), and were therefore much more likely to have already developed at least one diabetic complication. There is evidence of correlation between diabetes duration and deterioration in quality of life [43–45], mainly due to the development of complications. However, only a very small number of the studies included in this review covered the effect of the team intervention on the development of complications. Only eight of the studies (18%) measured nephropathy parameters, and none of these studies showed an improvement following the establishment of the team.

#### Team characteristics

Team compositions have been shown to be highly heterogeneous, with team-members coming from a variety of professional, educational and personal backgrounds. This heterogeneity of caregivers is likely to be of benefit in meeting the needs of highly heterogeneous patient populations who require individualised care approaches [46].

Unfortunately, most of the studies included in this review did not elaborate on the type of collaboration that occurred within the teams. Research on teamwork is complicated by inconsistent terminology, which is often used interchangeably and does not necessarily reflect the level of collaboration [47, 48].

Most studies included in this review failed to specify whether the different providers shared a team identity or code of conduct, nor did they report the presence of a supervising team leader. Previous research has shown that teams with a recognised leader appear to be more effective than teams without a leader [49, 50] but further research is needed to analyse the effects and the qualifications and skills required in a team leader. The question of whether a team leader should be a physician or a non-physician should also be addressed.

Team size and the relative proportions of physicians and non-physicians in the team did not seem to influence the outcome parameters. These findings suggest that there must be other parameters that are of importance when it comes to the effectiveness of a team. Communication between team-members seems to play an important role in team effectiveness, as it encourages the development of trust and mutual respect [51]. Also, effective teams need to consist of motivated, committed and experienced staff [52]. Interestingly, not a single study in this review covered the individual motivation and commitment of the physician and non-physician participants.

While randomised, controlled trials are widely considered the gold standard in medical science, other types of study designs, such as before/after studies, might be more beneficial when analysing psychological factors relating to task delegation and teamwork.

The main focus of this scoping review was on task delegation rather than the substitution of medical tasks. The definitions of these terms vary depending on the specific healthcare system and legal context in question. Previous research has shown the huge potential of the substitution of medical tasks to non-physician healthcare providers [19]. The findings of this review suggest that, when appropriately qualified and trained, non-physician team-members can probably achieve equal or even better health outcomes for diabetes patients than those achieved by physicians.

### Limitations

Several factors may affect the findings of this review and must be considered as potential limitations: 1) The lack of specific keywords and consistent terminology for describing task delegation and team interventions complicated the literature search, which may have resulted in relevant studies being missed. However, in order to minimise this limitation, a very extensive literature search method was conducted over a period of 12 months. 2) Due to the heterogeneity of identified studies, it was not possible to conduct a meta-analysis of results as part of this review. Instead, the review offers a description of the interventions and outcomes, and reveals gaps in existing research. 3) As in any systematic review, the findings of this review rely on the quality of the studies included in it, which appeared to be heterogeneous. Many of the studies included in the review maintained a high scientific standard and gave a detailed description of the methodology they used. However, there were also some studies of lesser quality that did not indicate basic methodological aspects, such as eligibility criteria, power and sample size calculation, underlying national or international guidelines, or study limitations and potential bias.

4) Studies with negative results may be underrepresented in the review, resulting in biased effects. 5) Given the differences in the expertise and training of non-physician healthcare providers, the legal backgrounds and the incentive systems in different healthcare systems around the world, the implications of this review for practice are limited. Recent study results reveal the potential of delegation and telemedicine in Germany [53], but further research is needed to justify task delegation in practice.

## Conclusion

The findings of this scoping review suggest that appropriately qualified and trained non-physicians could provide equal or possibly even improved diabetes care compared to that provided by physicians. The potential applications of task delegation are broad, and vary depending on the setting and patient population. Telemedicine might prove a helpful tool when implementing task delegation in practice. Given the vast differences in healthcare systems around the world, it is not possible to draw conclusions regarding the level of qualification and training required for non-physicians. As a result, implications regarding practice should be based on national rather than international study results. Further research should indicate precisely the adherence to evidence-based guidelines and the training and expertise of non-physicians in order to facilitate international comparisons.

Future studies should address whether task delegation could not only provide equivalent glycaemic control, but also lead to long-term improvements, and even effectively improve primary endpoints rather than surrogate parameters. Diabetes management is not solely based on glycaemic control, but rather implies comprehensive healthcare involving regular exams of different organ systems, from head to toe. As such, future research should place greater emphasis on the effects of task delegation on patient-centred parameters, as well as the prevention and early detection of diabetic complications. This review revealed an inconsistency between biochemical outcome parameters, such as HbA1c, and patient-centred outcome parameters, such as quality of life scores. Future research that effectively targets quality of life is needed in order to find out whether this inconsistency really exists.

Future studies of team-based diabetes care should give more attention to the assessment of team characteristics, especially the ideal team size and the involvement of physicians in the team. Related to this is the question of whether non-physician clinicians need to be supervised by physicians, or whether they should work independently.

While the importance of motivated, committed staff is undeniable, research on the individual characteristics of team members is scarce. Study designs other than randomised controlled trials might be of benefit.

## Supporting information

S1 File Search strategy. (DOCX)

S2 File PRISMA-Scr checklist. (DOCX)
