# Supplementary material for: Does task delegation to non-physician health professionals improve quality of diabetes care? Results of a scoping review
Source: PLoS One. 2019 Oct 11;14(10):e0223159. doi: 10.1371/journal.pone.0223159 (PMC6788697; doi:10.1371/journal.pone.0223159)
Supplement: S1 File — (DOCX) [file pone.0223159.s001.docx]

**Supporting information file: Search strategy**

The initial and updated literature searching was conducted in several databases (MEDLINE, EMBASE, Google Scholar) with a combination of different keywords. The keyword “diabetes” was used in combination with one keyword from group 1 and one keyword from group 2.

| Keyword from group 1:  Hospital  Structured Care  Managed Care  Delegation  Substitution | Keyword from group 2:  Nurse  Team  Dietitian  Pharmacist  Community Health Worker  Social Worker |
| --- | --- |

The following table shows the results of the different keyword combinations in the database “MEDLINE” for all studies published from 01/01/2014 – 12/31/2017 with the restriction “clinical trial”.

| ***Diabetes*** | | **KEYWORD GROUP 1** | | | | | **Total** |
| --- | --- | --- | --- | --- | --- | --- | --- |
|  |  | ***Hospital*** | ***„Structured Care“*** | ***„Managed Care“*** | ***Delegation*** | ***Substitution*** |  |
| **KEYWORD GROUP 2** | ***Nurse*** | ***79*** | 2 | 1 | 0 | 1 | 83 |
|  | ***Dietitian*** | ***15*** | 0 | 0 | 0 | 0 | 15 |
|  | ***Pharmacist*** | ***22*** | 0 | 0 | 0 | 0 | 22 |
|  | ***„Community Health Worker“*** | ***10*** | 0 | 0 | 0 | 0 | 10 |
|  | ***„Social Worker“*** | ***2*** | 0 | 0 | 0 | 0 | 2 |
|  | ***Team*** | ***88*** | 0 | 2 | 0 | 2 | 92 |
| **Total** | | 216 | 2 | 3 | 0 | 3 | **224** |

**Detailed search strategy criteria for the database MEDLINE:**

**(first column of the table above)**

(("diabetes mellitus"[MeSH Terms] OR ("diabetes"[All Fields] AND "mellitus"[All Fields]) OR "diabetes mellitus"[All Fields] OR "diabetes"[All Fields] OR "diabetes insipidus"[MeSH Terms] OR ("diabetes"[All Fields] AND "insipidus"[All Fields]) OR "diabetes insipidus"[All Fields]) AND ("hospitals"[MeSH Terms] OR "hospitals"[All Fields] OR "hospital"[All Fields]) AND ("nurses"[MeSH Terms] OR "nurses"[All Fields] OR "nurse"[All Fields])) AND (Clinical Trial[ptyp] AND ("2014/01/01"[PDAT] : "2017/12/31"[PDAT]))

(("diabetes mellitus"[MeSH Terms] OR ("diabetes"[All Fields] AND "mellitus"[All Fields]) OR "diabetes mellitus"[All Fields] OR "diabetes"[All Fields] OR "diabetes insipidus"[MeSH Terms] OR ("diabetes"[All Fields] AND "insipidus"[All Fields]) OR "diabetes insipidus"[All Fields]) AND ("hospitals"[MeSH Terms] OR "hospitals"[All Fields] OR "hospital"[All Fields]) AND ("nutritionists"[MeSH Terms] OR "nutritionists"[All Fields] OR "dietitian"[All Fields])) AND (Clinical Trial[ptyp] AND ("2014/01/01"[PDAT] : "2017/12/31"[PDAT]))

(("diabetes mellitus"[MeSH Terms] OR ("diabetes"[All Fields] AND "mellitus"[All Fields]) OR "diabetes mellitus"[All Fields] OR "diabetes"[All Fields] OR "diabetes insipidus"[MeSH Terms] OR ("diabetes"[All Fields] AND "insipidus"[All Fields]) OR "diabetes insipidus"[All Fields]) AND ("hospitals"[MeSH Terms] OR "hospitals"[All Fields] OR "hospital"[All Fields]) AND ("pharmacists"[MeSH Terms] OR "pharmacists"[All Fields] OR "pharmacist"[All Fields])) AND (Clinical Trial[ptyp] AND ("2014/01/01"[PDAT] : "2017/12/31"[PDAT]))

(("diabetes mellitus"[MeSH Terms] OR ("diabetes"[All Fields] AND "mellitus"[All Fields]) OR "diabetes mellitus"[All Fields] OR "diabetes"[All Fields] OR "diabetes insipidus"[MeSH Terms] OR ("diabetes"[All Fields] AND "insipidus"[All Fields]) OR "diabetes insipidus"[All Fields]) AND ("hospitals"[MeSH Terms] OR "hospitals"[All Fields] OR "hospital"[All Fields])) AND "Community Health Worker"[All Fields] AND (Clinical Trial[ptyp] AND ("2014/01/01"[PDAT] : "2017/12/31"[PDAT]))

(("diabetes mellitus"[MeSH Terms] OR ("diabetes"[All Fields] AND "mellitus"[All Fields]) OR "diabetes mellitus"[All Fields] OR "diabetes"[All Fields] OR "diabetes insipidus"[MeSH Terms] OR ("diabetes"[All Fields] AND "insipidus"[All Fields]) OR "diabetes insipidus"[All Fields]) AND ("hospitals"[MeSH Terms] OR "hospitals"[All Fields] OR "hospital"[All Fields])) AND "Social Worker"[All Fields] AND (Clinical Trial[ptyp] AND ("2014/01/01"[PDAT] : "2017/12/31"[PDAT]))

(("diabetes mellitus"[MeSH Terms] OR ("diabetes"[All Fields] AND "mellitus"[All Fields]) OR "diabetes mellitus"[All Fields] OR "diabetes"[All Fields] OR "diabetes insipidus"[MeSH Terms] OR ("diabetes"[All Fields] AND "insipidus"[All Fields]) OR "diabetes insipidus"[All Fields]) AND ("hospitals"[MeSH Terms] OR "hospitals"[All Fields] OR "hospital"[All Fields]) AND team[All Fields]) AND (Clinical Trial[ptyp] AND ("2014/01/01"[PDAT] : "2017/12/31"[PDAT]))
